# 

**Published:** 1868-05

**Authors:** 


### Books and Pamphlets Received.

Therapeutics and Materia Medica. A systematic Treatise on the Action and Uses of Medicinal Agents, including their Description and History. By Alfred Stillé, M. D., Professor of the Theory and Practice of Medicine and of Clinical Medicine in the University of Pennsylvania, etc., etc. Third Edition. Revised and Enlarged. In two volumes. Vols. 1 and 2. Philadelphia: Henry C. Lea; 1868. For sale by Theodore Butler.

On the Diseases of the Skin; A System of Cutaneous Medicine. By Erasmus Wilson, F. R. S. Seventh American, from the Sixth Revised English Edition. With twenty Plates and Engraving on Wood. Philadelphia: Henry C. Lea; 1868. For sale by Theodore Butler.

The Indigestions; or Diseases of the Digestive Organs Functionally Treated. By Thomas King Chambers, Honorary Physician to H. R. H., the Prince of Wales, etc., etc. Second American, from the Second and Revised English Edition. Philadelphia: Henry C. Lea; 1868. For sale by Theodore Butler.

Materia Medica for the Use of Students. By John B. Biddle, M. D., Professor of Materia Medica and General Therapeutics in the Jefferson Medical College, etc. Third Edition Enlarged, with Illustrations. Philadelphia: Lindsay & Blakiston; 1868. For sale by Theodore Butler.

Atlas of Venereal Diseases. By A. Cullerier, Surgeon to the Hospital Du Dieu, etc., etc. Translated from the French with Notes and Additions, by Freeman J. Bumstead, M. D., Professor of Venereal Diseases, in the College of Physicians and Surgeons, New York. With about one hundred and fifty beautifully colored figures, on twenty-six plates. Philadelphia: Henry C. Lea; 1868. Parts 2 and 3. For sale by Theodore Butler.

The Neuroses of the Skin: Their Pathology and Treatment. By Howard F. Damon, A. M., M. D. Philadelphia: J. B. Lippencott & Co.; 1868. For sale by Breed, Lent & Co.

Odontalgia, commonly called Tooth-Ache. Its Causes, Prevention and Cure. By Parsons Shaw. Philadelphia: J. B. Lippencott & Co.; 1868. For sale by Breed, Lent & Co.

Cases of Ovariotomy, by Warren Green, M. D.

